# Synthesis of a Graphene-Encapsulated Fe_3_C/Fe Catalyst Supported on Sporopollenin Exine Capsules and Its Use for the Reverse Water–Gas Shift Reaction

**DOI:** 10.1021/acssuschemeng.3c00495

**Published:** 2023-10-21

**Authors:** Waqas Malik, Jorge Pavel Victoria Tafoya, Szymon Doszczeczko, Ana Belen Jorge Sobrido, Vasiliki K. Skoulou, Andrew N. Boa, Qi Zhang, Tomas Ramirez Reina, Roberto Volpe

**Affiliations:** † School of Engineering and Materials Science, Queen Mary University of London, Mile End Campus, E1 4NS London, U.K.; ‡ Department of Chemistry, University of Hull, Hull HU6 7RX, U.K.; § Department of Chemical and Process Engineering, University of Surrey, Guildford, Surrey GU2 7XH, U.K.

**Keywords:** sporopollenin, iron, graphitization, catalyst support and pyrolysis

## Abstract

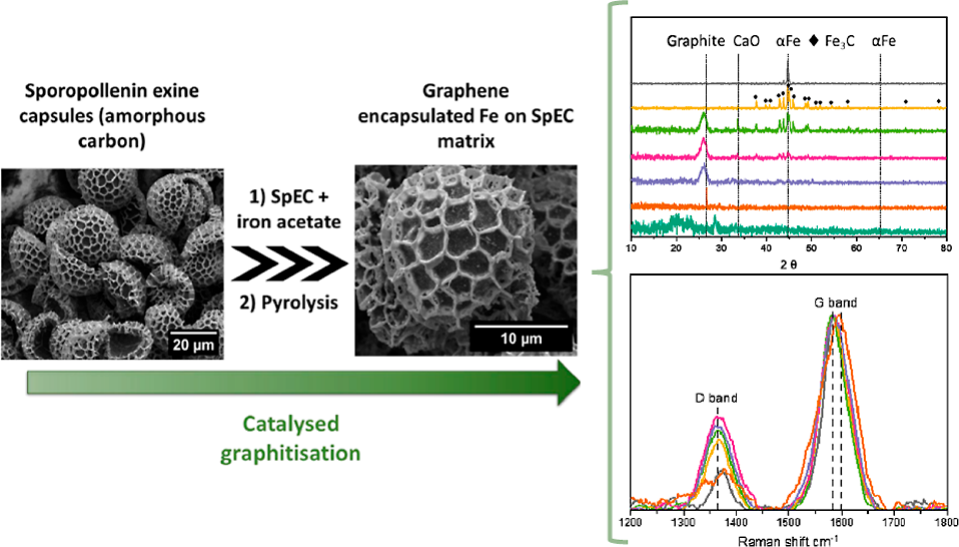

Bioderived materials have emerged as sustainable catalyst supports for several heterogeneous reactions owing to their naturally occurring hierarchal pore size distribution, high surface area, and thermal and chemical stability. We utilize sporopollenin exine capsules (SpECs), a carbon-rich byproduct of pollen grains, composed primarily of polymerized and cross-linked lipids, to synthesize carbon-encapsulated iron nanoparticles via evaporative precipitation and pyrolytic treatments. The composition and morphology of the macroparticles were influenced by the precursor iron acetate concentration. Most significantly, the formation of crystalline phases (Fe_3_C, α-Fe, and graphite) detected via X-ray diffraction spectroscopy showed a critical dependence on iron loading. Significantly, the characteristic morphology and structure of the SpECs were largely preserved after high-temperature pyrolysis. Analysis of Brunauer–Emmett–Teller surface area, the D and G bands from Raman spectroscopy, and the relative ratio of the C=C to C–C bonding from high-resolution X-ray photoelectron spectroscopy suggests that porosity, surface area, and degree of graphitization were easily tuned by varying the Fe loading. A mechanism for the formation of crystalline phases and meso-porosity during the pyrolysis process is also proposed. SpEC-Fe10% proved to be highly active and selective for the reverse water–gas shift reaction at high temperatures (>600 °C).

## Abstract 2

Sporopollenin exine capsules: a bioderived, sustainable material for an iron-catalyzed reverse water–gas shift reaction.

## 1 Introduction

Catalyst supports are pivotal for the effective utilization of metal-based catalytic species in numerous heterogeneous reactions. Porous carbon-based catalyst supports have thus far been popular among both researchers and industry alike, proving indispensable in gas-phase reactions such as Fischer–Tropsch synthesis, as well as electrocatalytically driven reactions, like dioxygen reduction or hydrogen evolution reactions.^1^ Activated carbon materials possess compelling properties for catalyst support; they may be easily prepared with a high surface area and tunable pore size distribution, are highly electrically conductive, and display high chemical and thermal stability.^2,3^ These are most frequently prepared via high-temperature pyrolysis of organic precursors, those being either synthetic in nature, such as carbon black derived from waste tires, or are biomass-based.^4,5^

Biomass has shown great potential to replace conventional synthetic supports for catalysts and to act as an alternative carbon-rich precursor, as it may be abundant, cheap, sustainable, and renewable.^6−8^ Some activated carbons derived from biomass develop a trimodal pore structure, with the preserved pre-existing macropore structure serving to enhance reactant mass transfer to the metallic active sites during catalytic reactions. An excellent example is the many pollen and spore grains, which show a uniform, three-dimensional (3D) macrostructure. They are composed of a core containing genetic and plasmic material protected by an extraordinarily strong double shell, which consists of an inner layer (intine) and an outer layer (exine). The intine is mainly composed of delicate cellulose, hemicellulose, and pectin, while the outer layer, or exine, is comprised of the biopolymer sporopollenin (Sp), which is a mechanically and chemically resilient, cross-linked organic polymer. Sp has been described as one of the toughest organic compounds of natural biological origin.^9^ Sp can be separated from the intine and extracted to obtain Sp exine capsules (SpEC). Valuable properties of SpECs such as porous morphology and extreme uniformity in size distribution, high thermal stability, as well as being stable toward chemical attacks (e.g., strongly acidic or alkaline conditions) could make them an ideal catalyst support. Modified SpECs have mainly been applied as adsorbents for various analytes, including dyes,^10^ metals,^11,12^ pesticides,^13^ and drugs.^14,15^ SpECs have also been utilized as a solid support for peptide synthesis.^16^ To the best of our knowledge, however, only one study has reported the utilization of SpECs as a support for metal catalysts.^17^ Baran and co-workers^17^ used modified SpECs as a support for a palladium(II) catalyst for a Suzuki coupling reaction. In that work, the metal is not directly bound to (interacting with) the SpECs, which are used as a solid support; instead, it is bound to the metal via a linker and chelate group using a complex modification. Their catalyst exhibited a remarkable turnover number and turnover frequency as high as 40,000 and 400,000, respectively, indicating high stability and reusability. This was credited to the ease of modification of Sp and its high thermal and mechanical stability.

Iron (Fe) is a very versatile catalyst and is especially suited for green energy-based catalysis due to its ability to function under a wide range of temperatures and H_2_/CO ratios, being cheap and abundant.^18^ Fe-based catalysts have been demonstrated to be a great alternative to noble metals in relevant reactions such as oxygen reduction,^19^ direct coupling of methane and conversion into ethylene,^20^ and hydrogenation of nitroarenes.^21^ Conversely, further applications of Fe catalysts are hindered by features like easy deactivation by oxidation and agglomeration of the catalyst.^22^ To prevent this and increase its chemical stability, encapsulation of the metal particles by a protective layer has been proven to be an effective solution^23^ to the deactivation problem of conventional Fe catalyst systems.

Carbon-encapsulated Fe nanoparticles (CEINP) have been produced by various methods, including chemical vapor deposition,^24^ arc discharge,^25^ and combustion/detonation.^26^ However, these methods involve using carbon precursors that require significant amounts of energy to produce, such as toluene, acetone, glucose, and graphite. Iron has been well documented to catalyze the graphitization of amorphous carbons during high-temperature treatment.^27,28^ This allows Fe particles to be encapsulated within a graphitized carbon shell, thus prolonging the life of the catalyst by protecting the iron core from oxidation. Ding et al.^29^ suggested a growth model to explain the mechanism of the formation of CEINP via simple impregnation and heat treatment. They proposed that the graphitic layers/shells are produced as the dissolved carbon atoms in the Fe carbide precipitate onto the particle surface. Even though research has been done on synthesizing CEINP, the tailoring of porosity, surface area, morphology, and degree of graphitization becomes particularly difficult when the starting material is raw biomass instead of their purified derivatives like lignin or cellulose. Typically, three different macromolecules (lignin, cellulose, and hemicellulose) make up raw biomass together with a small fraction (typically a few %w/w) of inorganics.^30^ Neeli and Ramsurn^31^ compared the synthesis and formation mechanisms of Fe nanoparticles in graphitized carbon matrices using biochar from different model biomass compounds (lignin, cellulose, and hemicellulose) as support. They were able to explain the formation of different phases observed in carbon-encapsulated Fe composites. They also showed that lignin-like model compounds formed somewhat different composites than cellulose and hemicellulose. As the structure of SpECs is quite different from that of lignin and polysaccharide polymers,^32^ further research is required to develop an efficient method of synthesizing sustainable CEINP using SpEC biomass as the carbon source. Furthermore, discovering how to utilize SpECs as carbon support for Fe particles while retaining the original, desirable morphology during impregnation and high-temperature carbonization warrants exploration.

In this study, a demonstration of a simple, cheap, and relatively green pyrolysis strategy for the synthesis of sustainable mesoporous CEINP using SpECs biomass as the carbon support was investigated. Additionally, the effect of the concentration of the iron precursor on the catalyst formation is reported. Details of the formation mechanism of CEINP and what effect the concentration of Fe has on the physicochemical properties of synthesized CEINP are explained through the obtained results from multiple characterization techniques of the raw SpECs, carbonized SpECs, and the CEINP obtained from the pyrolysis of Fe-loaded SpECs. We also report that the synthesized iron-based catalyst supported on SpECs (SpEC-Fe10%) demonstrated both high activity and selective for the reverse water–gas shift (RWGS) reaction. The effective catalytic performance, paired with sustainable methodology and low production costs, establishes that SpECs are a capable alternative to not only conventional synthetic catalyst supports but also typical lignocellulosic biomass.

## 2 Experimental Section

### 2.1 Materials

SpECs used in this study were obtained from raw spores by extraction. In brief, the extraction was conducted by heating 6% sodium hydroxide for 24 h, as previously reported.^33^ The resulting SpECs are monodispersed, with an almost uniform size of approximately 27 μm in diameter hollow carbonaceous particles, and are almost perfectly spherical in shape. Fe(II) acetate powder (99.9% purity) and methanol were obtained from Sigma-Aldrich.

### 2.2 Synthesis Method

Six different solutions of iron(II) acetate (Fe(CO_2_CH_3_)_2_, Fe(OAc)_2_) in methanol (20 mL) were prepared with an increasing weight percentage of Fe(OAc)_2_/(SpECs + Fe(OAc)_2_). The weight percentages of Fe(OAc)_2_ used to prepare the impregnation solutions were 0, 1, 5, 10, 20, and 50%, respectively.

Samples were prepared by adding SpECs to each of the Fe(OAc)_2_ solutions, forming a dark green/brown suspension. The resulting mixture was stirred for 24 h at ambient temperature, followed by drying for 24 h at 40 °C in the air. Once dried, the samples were pyrolyzed at 1000 °C for 1 h in a tubular furnace under N_2_ flow and cooled to room temperature. The untreated SpEC sample was denoted simply as SpEC and the treated samples were denoted as SpEC-Fe*x*, where *x* is the weight percentage of FeAc used.

The amount of Fe(OAc)_2,_ the molarity of the solution, and the yield of the product formed in each sample are shown in Table 1. The yield of the final product was calculated using (eq 1).
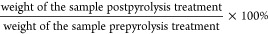
1

**Table 1:** Fe(OAc)_2_ Concentration and Its Effect on the Yield (% w/w) of the SpEC-Fex Synthesized

sample	Fe(OAc)_2_ (mg)	Fe(OAc)_2_ solution conc. (mM)	yield (% w.w)
SpEC-Fe0%	—	—	16.5
SpEC-Fe1%	4.04	1.2	20.2
SpEC-Fe5%	21.1	6.1	22.4
SpEC-Fe10%	44.4	12.8	23.3
SpEC-Fe20%	100.0	28.8	24.8
SpEC-Fe50%	400.0	115.0	26.5

### 2.3 Physical and Chemical Characterization Techniques

Elemental analysis of the extracted SpECs was performed using an EA-1108 CHNS Elemental Analyzer (Fisons). The elemental analysis of extracted SpECs indicated that they were made from mainly C: 57.45% w.w., H: 9.66% w.w., and N:1.45% w.w.

SpECs and the synthesized SpEC-Fe*x* were characterized by the following techniques. X-ray diffraction (XRD) was used to investigate crystallinity and structural information on the samples using a Siemens D5000 powder diffractometer with aq/2q geometry in reflection mode equipped with a Cu α source with a wavelength of 1.54 Å. Field emission scanning electron microscopy (SEM, FEI InspectF50) equipped with an energy dispersive spectrometer (EDS) was operated at an accelerating voltage of 15 kV and a spot size of 5 to explore the morphology and surface structure of materials. X-ray photoelectron spectroscopy (XPS, Thermo Scientific, Nexsa) was used to investigate the chemical composition of the samples. For further structural and chemical analysis, Raman spectroscopy (Renishaw in Via, 442 nm) was employed to obtain the defect/graphitic ratio (*I*_D_/*I*_G_) and analyze how the change in Fe concentration affected the degree of graphitization. Finally, the surface area measurements (Brunauer–Emmett–Teller—BET) were conducted using N_2_ as an adsorbate in a Nova Quantachrome instrument.

### 2.4 Catalytic Characterization Techniques

The RWGS reactions were carried out and recorded in a vertical continuous fixed bed reactor. An ABB AO2020 Advanced Optima Process Gas Analyzer was used for the online analysis of reactants and products (CO and CH_4_). The reactor was a 7 mm inner diameter quartz tube in which 0.25 g of catalyst was placed on quartz wool placed in the middle of the reactor. The sample was heated in flowing N_2_ from room temperature to 350 °C. Then, the N_2_ was replaced by the feed gas mixture of H_2_/CO_2_ = 4:1 at a constant weight hourly space velocity (WHSV) of 12,000 mL g^–1^ h^–1^, and the products of the reaction were evaluated from 350 to 750 °C. At each temperature, the gas products were analyzed after 20 min of steady–state reaction.

CO_2_ conversion (eq 2), CO selectivity (eq 3), and CH_4_ selectivity (eq 4) were measured based on the above tests. The relative experimental error in CO_2_ conversion and CO/CH_4_ selectivity in this work was given within ±0.5%. Where  is the inlet molar flow (kmol/min) of CO_2_ in the reactant mixture and  are the outlet molar flows in the product stream of CO, CH_4_, and CO_2_, respectively.
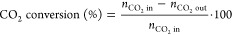
2
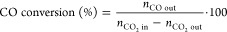
3
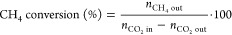
4

## 3 Results and Discussion

### 3.1 Synthesis of SpEC-Fex Catalysts by the Carbothermal Reduction Process

The results of the synthesis method described above are listed in Table 1. The results show a clear correlation between the Fe(OAc)_2_ concentration and the yield of the product, where a higher concentration of Fe(OAc)_2_ leads to a higher product yield. This is simply due to more Fe being deposited onto the surface of the SpECs during the impregnation step. The natural carbon support (SpECs) is consumed as a reducing agent during the carbothermal reduction reaction, where Fe oxide particles are reduced to iron carbide and/or metallic iron.

### 3.2 XRD Crystallographic Characterization

**Figure 1:**
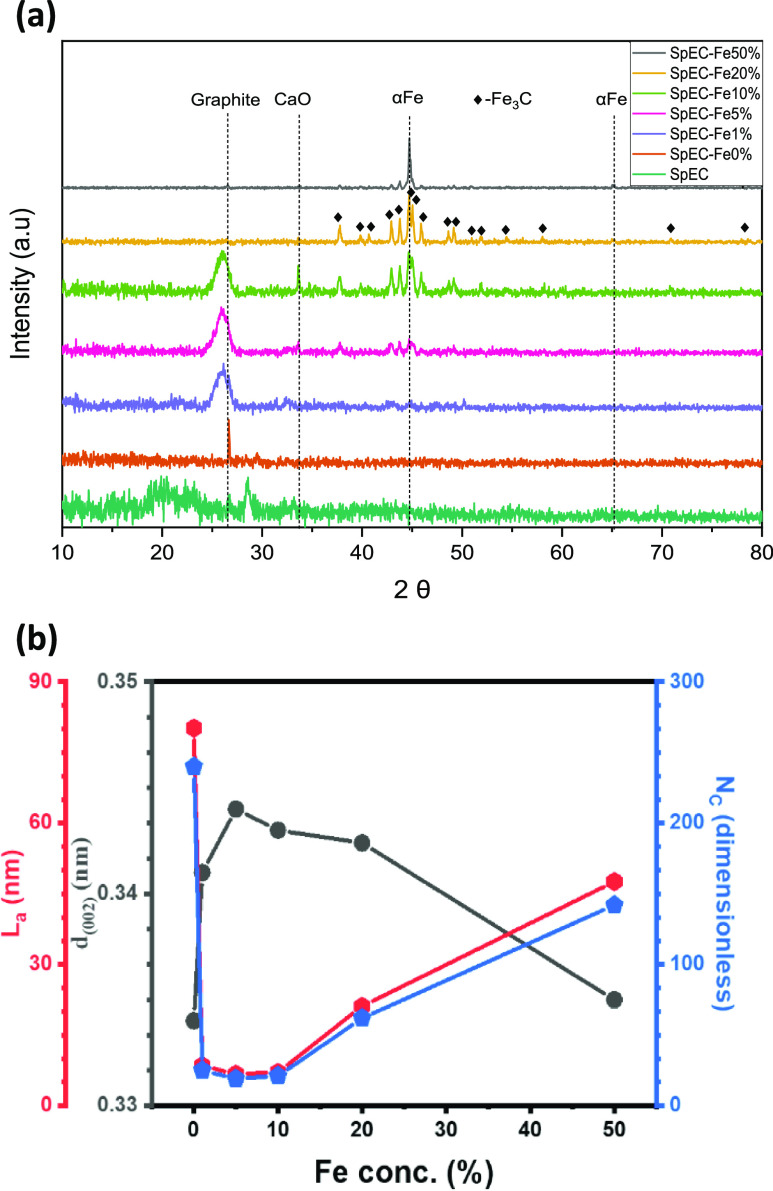
X-ray characterization: (a) diffraction patterns and (b) crystallographic calculated properties of the obtained materials, where *d* represents the interlayer spacing, *L*_a_ represents the crystallite size, and *N*_c_ represents the number of layers.

Figure 1 displays the XRD spectra of the untreated SpECs and the SpEC-Fe*x* samples. The absence of a graphite peak was expected in the untreated SpEC, as it is a carbonaceous matrix of a nongraphite carbon that mainly comprises amorphous carbon in the form of phenolic compounds, long-chain aliphatic compounds, and polyvinyl derivatives.^34^ It is worth noting that the presence of phenylpropane in the structure of Sp, differentiates it from the lignin or lignin-like compounds.^35^ This is further supported by the typical broad, amorphous characteristic peak at 2θ = 19.48°.

The diffraction peak at 2θ of 26.5° is present in all samples that were pyrolyzed at 1000 °C and represents the graphite (002) plane. The SpEC-Fe*x* samples produced much broader graphite peaks compared to the SpEC-Fe0%, which gave a much sharper peak. These layers of graphite found in CEINP have proven to shield the metal nanoparticles from oxidation.^36^ The intensity of the graphite peak is significantly lower in samples with higher Fe loading, particularly in SpEC-Fe20% and SpEC-Fe50%. However, this may not be accurate as the X-rays can travel right through some of the thin graphitic layers, causing the graphitic phase to be underestimated, especially when a high concentration of Fe is present. In reality, the graphite peak was found to be slightly sharper with an increase in the concentration of Fe. These results are in line with previous ones, which have also shown that only a small quantity of Fe is needed to initiate graphitization in lignocellulosic-based biomass.^28^

For further investigation of the enhancement of graphitization and formation of few-layered graphene-alike domains, Bragg’s and Sherrer’s equations were used to determine the change of interlayer spacing (*d*) and crystallite size (*L*_a_) and estimate the number of layers (*N*_c_) (Figure 1b). It was found that the optimal regime of concentration of Fe(OAc)_2_ is between 5 and 10% to induce the formation of few-layered graphene-like domains. It is clear that at that regime, the samples show closer *d* spacing and crystallite size values to those predicted for graphene-like materials. The decrease in the crystallite size implies an enhancement of surface area as well as the number of graphitic layers formed during thermal treatment. Interestingly, the sample without Fe(OAc)_2_ shows graphitization regardless of the presence or lack of Fe to catalyze the graphitization reaction during thermal treatment. Nonetheless, it is well-known that thermal treatment of amorphous carbon materials can lead to the formation of graphitic domains.^37,38^ The extent of graphitization in the SpEC-Fe*x* samples is discussed in more detail in Section 3.4.

The diffraction peaks at 2θ of 44.7 and 65.1° corresponded to the body-centered cubic (bcc) α-Fe (110) and (200) planes and were found for all the samples loaded with Fe(OAc)_2_. As expected, the intensity of these two peaks was found to be the lowest in SpEC-Fe1%, followed by SpEC-Fe5%, as the concentration of Fe(OAc)_2_ was the lowest in these two samples, respectively. The sharpening of the peak and an increase in intensity suggest that larger Fe particles were formed. However, samples SpEC-Fe10, 20, and 50% displayed similar intensities even though the concentration of Fe(OAc)_2_ was successively increased.

The diffraction peaks at 2θ of 37.6, 37.8, 39.8, 40.7, 42.6, 43.8, 44.6, 45.0, 45.9, 48.4, 49.1, 51.8, 54.4 and 57.9, 70.9, 77.9, and 78.6° represent cementite (θ-Fe_3_C), and correspond to crystallographic planes of (121), (210), (002), (201), (211), (102), (220), (031), (112), (131), (221), (122), (230), (301), (301), (123), and (133), respectively. Fe_3_C peaks displayed a trend similar to that of α-Fe peaks, where an increase in Fe gave well-resolved and sharper peaks with increasing intensities, also indicating the formation of larger Fe_3_C particles. However, in this case, the intensity seemed to increase up to SpEC-Fe20%. The Fe_3_C peaks in sample SpEC-Fe50% seemed to have considerably lower intensities, while the α-Fe peaks remained unchanged and dominant.

Calcium is naturally found in raw SpEC.^39^ As such, small traces of calcium were still present in untreated SpECs (Figure S1). Figure 1a shows clear peaks of calcium oxide (CaO) in Sp-Fe5% and Sp-Fe10%. Peaks with very low intensities are also present in SpEC-Fe20%. The CaO diffraction peaks are represented at 2θ of 33.6° and correspond to a crystallographic plane of (110). Interestingly, this peak was missing in the SpEC-Fe0% and SpEC-Fe1%, suggesting that the presence of Fe had some effect on the formation of CaO. In fact, after an increase in the intensity of the CaO peaks from SpEC-Fe5% to SpEC-Fe10%, the intensity of this peak decreases with an increase in the concentration of Fe, as indicated by low intensities in SpEC-Fe20% and virtually no presence in SpEC-Fe50%.

### 3.3 SEM Morphological Characterization

**Figure 2:**
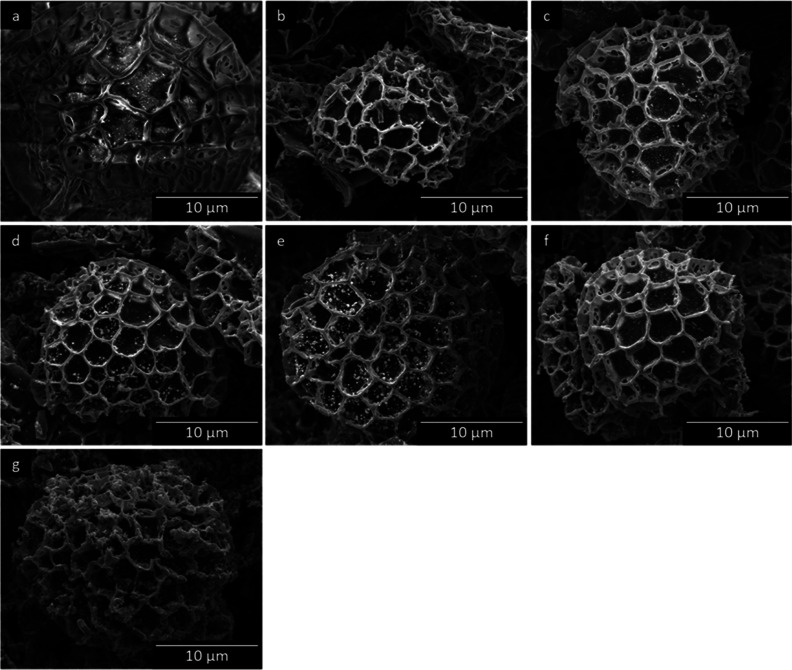
Surface morphology (SEM micrographs) of (a) untreated SpECs, (b) SpEC-Fe0%, (c) SpEC-Fe1%, (d) SpEC-Fe5%, (e) SECp-Fe10%, (f) SpEC-Fe20%, and (g) SpEC-Fe50%.

**Figure 3:**
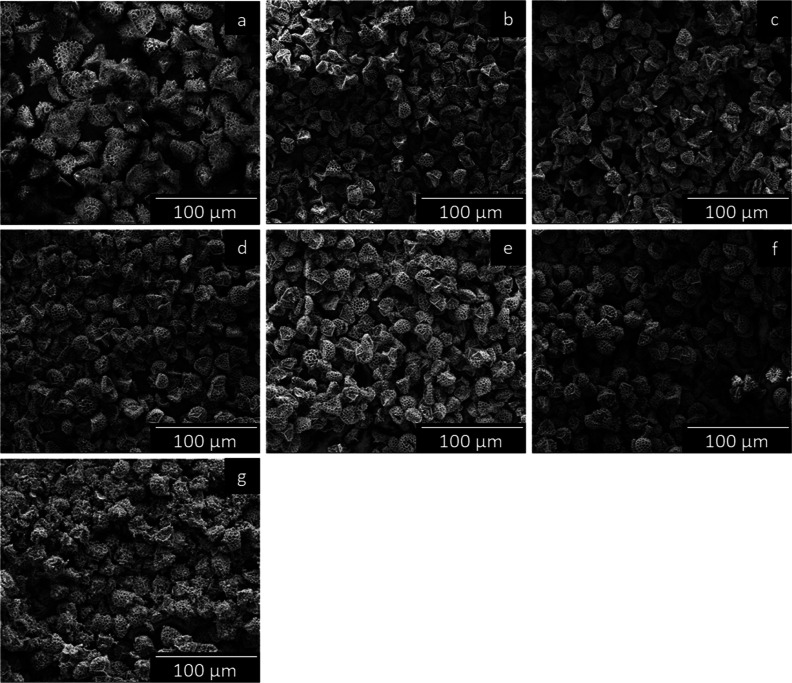
SEM micrographs of (a) untreated SpECs, (b) SpEC-Fe0%, (c) SpEC-Fe1%, (d) SpEC-Fe5%, (e) SpEC-Fe10%, (f) SpEC-Fe20%, and (g) SpEC-Fe50%.

SEM was employed to examine the morphology of the SpECs and help to determine whether SpECs could retain their surface structure after the high-temperature thermal treatment. Figures 2 and 3 show SEM images of individual particles and bulk untreated SpECs and SpEC-Fe*x* samples, respectively. SEM images of untreated SpECs (Figure 2a) reveal that the particles have a diameter of roughly 27 μm, and their characteristic morphology consists of interconnected uniform hexagonal-shaped microstructures resembling the shape of a honeycomb. The overall structure of untreated SpECs has the shape of a hemispherical cap, with a triplet structure (Y-shaped) on the underside of the particle, which is typical for this specific pollen species (Figure 3a). EDS analysis was conducted on the SpEC-Fe*x* composites (Figure S2), indicating that the Fe is well dispersed in all samples.

After the heat treatment, the size of SpECs decreased by 44%, from approximately 27 to 15 μm (Figure 2a–g) in diameter. This was due to the loss of volatiles from the carbon-rich support occurring from the high-temperature treatment, which led to an almost homogeneous shrinkage of the SpECs support volume. According to Figure 2a–f, SpEC-Fe0% and the SpEC-Fe1%–20% samples clearly retained the original almost spherical microstructure of their parent support: untreated SpECs, with their characteristic hexagonal honeycomb structure being replicated with noteworthy accuracy, even after the high-temperature treatment and Fe loading, respectively. Additionally, there was little or no evidence of any heavy residue accumulating on the surface of the particles. In Figure 2g, it can be seen that although the original hexagonal honeycomb shape of the SpECs support can be recognized to some degree, it is obvious that the shape of the SpECs synthesized using the highest concentrations of Fe(OAc)_2_ (50%) was lost and somewhat distorted compared to that of the untreated SpECs and all of the other samples. An increase in Fe concentration led to heavier residual accumulation on the surface of the particles, thus leading to more of the structure being lost compared to the original morphology. While individual particles with retained morphology were found in low quantities, the bulk images show a more complete picture of the morphology of the majority of the SpEC-Fe50% sample (Figure 3g), where most of the particles did not retain the original morphology. A small amount of aggregation and cluster formation was also observed. Overall, Figure 3a–f suggest that impregnation of SpECs with up to 20% Fe(OAc)_2_ by weight allows preservation of the original morphology, with no signs of major aggregation, residual accumulation, or breaking up of the individual particles, which is key to their potential engineering applications.

### 3.4 Raman Spectroscopy Analysis

**Figure 4:**
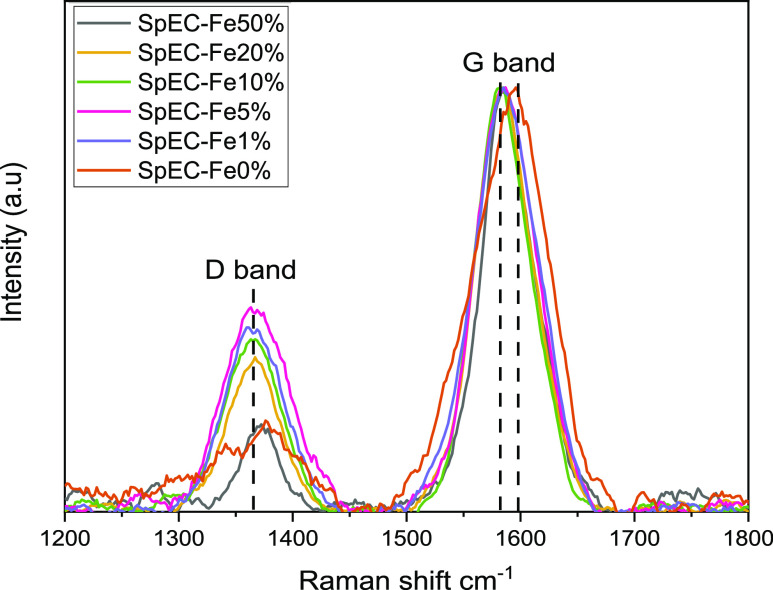
Raman spectra depicting the D and G bands of the SpEC-Fex composites.

Three distinct peaks were observed in all samples at approximately ∼1367, ∼1585, and ∼2740 cm^–1^, corresponding to the D, G, and G′ (also referred to as 2D) bands, respectively (Figure S3). The presence of D and G′ bands indicates a high concentration of amorphous carbon and disordered graphitic domains, rationally attributed to the nature of the initial biomass material. The G band is related to sp^2^-hybridized carbon in the 2D hexagonal lattice (E_2g_ vibrational mode), indicating the presence of ordered graphene and graphitic domains. The intensity ratio of the D and G bands (*I*_D_/*I*_G_ ratio) is used to quantify the defects of carbon-based material. A low *I*_D_/*I*_G_ ratio (relatively weaker D band) indicates a higher degree of graphitization,^40^ signifying a decrease in amorphous sp^2^-bonded carbon such as organic fragments, molecules, and functional groups. Figure 4 shows the D and G bands of all the samples after normalization. The intensity of the D band varies with each sample, while the intensity of the G band remains the same. Table 2 shows the *I*_D_/*I*_G_ ratio calculated from the areas of the D and G bands measured in the Raman spectra. Two deductions can be made from the *I*_D_/*I*_G_ ratio: first, an increase in Fe concentration leads to a lower *I*_D_/*I*_G_ ratio (from SpEC-Fe5% to SpEC-Fe50%), which is consistent with previous reports of Fe-catalyzed graphitization of amorphous carbon,^41^ second, the absence of Fe in sample SpEC-Fe0% resulting in a low *I*_D_/*I*_G_ ratio comparable to that of SpEC-Fe50%, which corroborates the XRD results. Similarly, the SpEC-Fe1% sample displayed a lower *I*_D_/*I*_G_ ratio than SpEC-Fe5%, closer to the SpEC-Fe10% ratio. These findings were in agreement with the results of Gai et al,^42^ who reported that the degree of graphitization of porous graphite carbon produced from biomass did not reduce when no or a very low concentration of Fe was used. The G peak of SpEC-Fe0% was slightly shifted from 1585 to 1596 cm^–1^ compared to all the other samples that were treated with Fe. A peak shift to a higher value, closer to 1600 cm^–1^, is consistent with the layering of the nanocrystalline graphitic domains.^40^ This is in agreement with the XRD characterization and crystalline properties determined for each sample discussed in Section 3.2.

**Table 2:** *I*_D_/*I*_G_ Ratio of SpEC-Fex Samples Indicating the Defects of Carbon-Based Materials

sample	SpEC-Fe0%	SpEC-Fe1%	SpEC-Fe5%	SpEC-Fe10%	SpEC-Fe20%	SpEC-Fe50%
*I*_D_/*I*_G_ ratio	0.14	0.39	0.48	0.40	0.30	0.10

The strong *G*′ peak (Figure S3) is a feature of the second order of zone-boundary phonons and is also a significant indication of a graphitic structure. The width and position of the peak provide information about layering and stacking of graphene sheets, where a single-layered graphene would be represented by a sharp *G*′ peak, while a few-layer or multilayer graphene would be represented by a much broader *G*′ peak. Overall, the Raman spectra (Figure S3) demonstrate that all samples formed well-graphitized few/multilayer graphene domains. This is further supported by the results discussed in Section 3.2.

### 3.5 BET Surface Area Analysis

**Figure 5:**
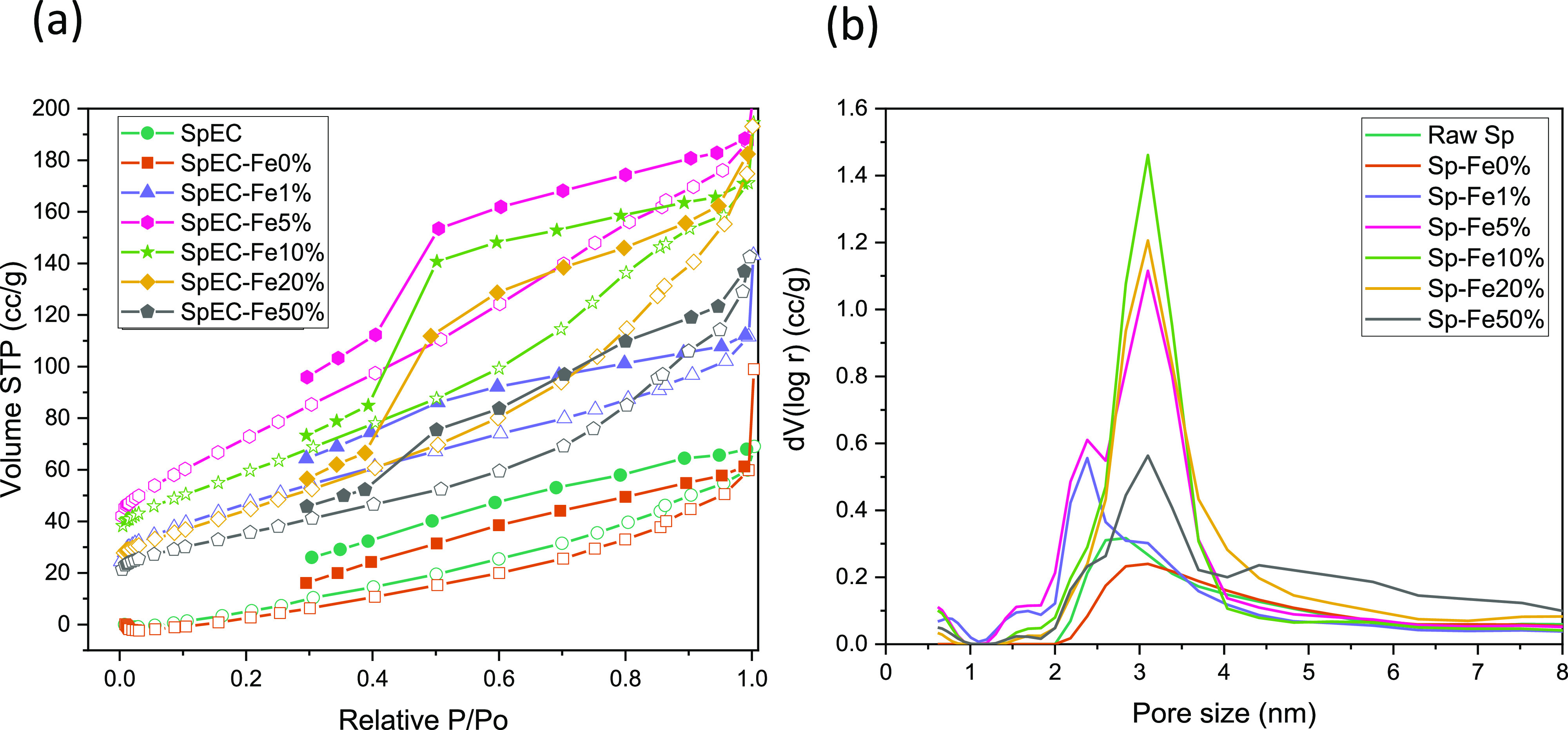
(a) Nitrogen adsorption–desorption isotherms of untreated SpEC and SpEC-Fex composites and (b) pore size distribution of untreated SpEC and SpEC-Fex composites.

The pore size distribution and textural characteristic properties of untreated SpECs and all Sp-Fe*x* samples are shown in Figure 5a,b and summarized in Table 3, respectively. According to the International Union of Pure and Applied Chemistry (IUPAC), all the samples display type IV adsorption–desorption isotherms (Figure 5a). The hysteresis loops of untreated SpECs belong to type H4, whereas SpEC-Fe0% and SpEC-Fe1% belong to a hybrid between H3 and H4, demonstrating that slit-shaped pores and a mixture of slit-shaped and wedge-shaped pores constitute most of the porous structure of the material. The main difference between the hysteresis loops is found on the less pronounced plateau in samples SpEC-Fe0% and SpEC-Fe1% at high P/P_0_ (0.9–1). The H4 and H3/H4 hybrid loops are usually represented by activated carbons and other nonporous absorbents.^43^ In contrast, hybrid H2 and H3 hysteresis loops were found in all samples containing Fe. The hysteresis loop in the relative pressure section of 0.4–0.9 occurred due to capillary condensation, demonstrating the existence of mesopores. The sharp closing of the hysteresis loop at the pressure region of approximately 0.4 is typically a result of “ink bottle” pores with a narrow vent and a large internal cavitation hole.^44^ This was present in all samples with Fe loading besides SpEC-Fe1%. As the amount of Fe loading is decreased (excluding SpEC-Fe0% and SpEC-Fe1%), the nitrogen isotherm at low *P*/*P*_0_ (0–0.4) increases slightly, while at high P/P_0_ (0.8–1) a more significant increase in nitrogen isotherm was observed. This indicates that the addition of the Fe component promotes a slight increase in the number of micropores and a larger increase in the number of macropores and mesopores.

**Table 3:** Textural Properties (Surface Area and Pore Volume) of Untreated SpEC and SpEC-Fex Samples

sample	surface area (m^2^ g^–1^)	pore volume (cm^3^ g^–1^)
untreated Sp	13	0.099
SpEC-Fe0%	32	0.087
SpEC-Fe1%	170	0.161
SpEC-Fe5%	263	0.274
SpEC-Fe10%	211	0.249
SpEC-Fe20%	162	0.251
SpEC-Fe50%	126	0.191

Pore size distributions were obtained via quenched solid density functional theory (QSDT). In previous literature, nonlocal density functional theory has generally been used to calculate pore size distribution, which presumes the carbon support surface to be geometrically and chemically smooth. On the other hand, QSDFT considers surface roughness in disordered carbons, thus delivering a far more accurate representation of the adsorption isotherm.^31^ The textural characteristics displayed in Table 3 show a trend similar to the nitrogen isotherms. Only a small change was observed between the untreated SpECs and SpEC-Fe0%, where the surface area increased only slightly from 13 to 32 m^2^ g^–1^, while the pore volume remained relatively the same. The surface area generally increased along with decreased Fe loading (excluding SpEC-Fe0% and SpEC-Fe1%). This was also true for pore volume, though the difference was not as evident between the SpEC-Fe10% and SpEC-Fe20%.

The average pore size was found to be 3 nm for all of the samples, with only one major peak being detected (Figure 5b). Small secondary and tertiary peaks were observed at the 1.6 and 2.3 nm marks in samples of SpEC-Fe1% and SpEC-Fe5% only, indicating the existence of micropores. The SpEC-Fe50% sample had the highest number of mesopores. These results are in accordance with the nitrogen isotherms discussed earlier in this section. The reason why untreated SpECs and SpEC-Fe0% had a lower surface area and pore volume compared to samples with higher concentrations of Fe was attributed to the catalytic gasification capability of the Fe.^45^ This phenomenon occurs due to the etching of the carbon matrix by redox reactions between amorphous carbon and metal oxide species. Additionally, the carbothermal reduction produces H_2_O, CO_2_, and other volatile gases, which participate to pore development also *via* gasification.^46^ From the results, it is also noticeable that a lower amount of Fe loading (excluding SpEC-Fe0% and SpEC-Fe1%) led to a larger amount of micropores, a higher surface area, and a larger pore volume, with the SpEC-Fe5% having the highest values among all the samples. This could be due to a few reasons, one of them being that the degree of graphitization induced in the carbon matrix by Fe particles during high-temperature pyrolysis treatment (1000 °C) breaks down the micropores in favor of mesopore formation.^47^ The existence of graphitic layers backed up by XRD (see Section 3.2) and Raman (see Section 3.4) spectra in all SpEC-Fe*x* samples, shows that, in general, a higher Fe loading leads to a higher degree of graphitization. Hence, a decrease in surface area is observed in samples with higher Fe loadings (Table 3). Furthermore, the Fe particles could potentially fill/block the carbon matrix pores, leading to the reduction of the surface area and pore volume.^48^ Accordingly, this is more apparent in samples with a higher Fe loading. The synergetic effect of these two outcomes helps explain the values shown by BET. In summary, a small increase in Fe concentration does increase the surface area due to catalytic etching. However, when a large amount of Fe is added, the surface area decreases. This is due to a combination of aggregation of Fe particles, which may lead to blockage of pores. Also, a higher Fe concentration is shown to increase the formation of graphene, which is usually associated with a decreased surface area in porous structures.

The values of surface area shown are appreciably lower than those reported for catalysts based on commercially available activated carbons, such as those used in,^49^ both for the fresh (1305 m^2^ g^–1^) and spent (628 m^2^ g^–1^) catalysts. A key point to note here is that, unlike most commercially available carbon-based supports (like activated carbon), we did not activate our biomass support anyway. This was done in order to solely investigate the interaction between iron and SpECs without adding factors affecting the final product.

### 3.6 XPS Surface Analysis

**Figure 6:**
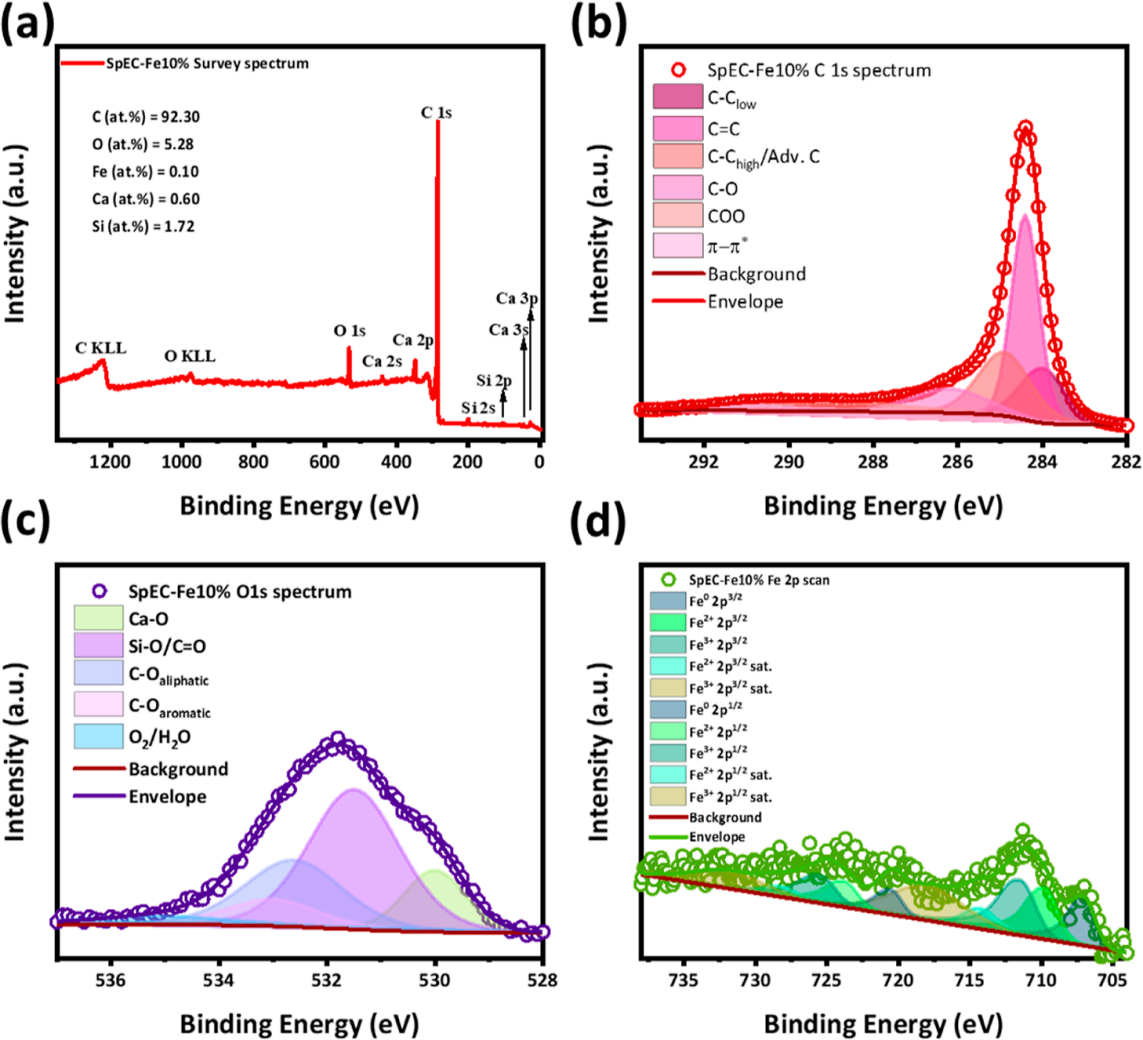
XPS analysis of the SpEC-Fe10% sample: (a) survey spectrum, (b) C 1s high-resolution spectrum, (c) O 1s high-resolution spectrum, and (d) Fe 2p high-resolution spectrum.

XPS was used to determine the average chemical environment on the surfaces of the different SpEC-Fe*x* samples. Figure 6 shows the analyzed survey, C 1s, O 1s, and Fe 2p spectra of the SpEC-Fe10% sample after thermal treatment (1000 °C) under an inert atmosphere (N_2_). Complementary XPS surface analysis was performed on the untreated SpECs and all other SpEC-Fe*x* samples (Figures S4–S8). It was found that the SpEC-Fe10% sample contains mainly C, O, Fe, Ca, and Si (Figure 6a), with an average atomic percentage of 92.30, 5.28, 0.10, 0.60, and 1.72%, respectively. Whereas for the other samples with different concentrations of Fe(OAc)_2_, the oxygen content remained higher. This indicates that for the SpEC-Fe10% sample, the reduction of oxygen functional groups in the presence of Fe(OAc)_2_ at this specific concentration meets an optimal point (Figures S4–S8). Additionally, Fe surface analysis on sample SpEC-Fe1% shows no superficial Fe species, indicating diffusion of iron through the carbonaceous matrix, which is backed by the presence of low-intensity diffraction peaks correlated to that of Fe°/Fe_3_C, observed in XRD diffractograms (Figure 1a). This phenomenon can be reasonably explained due to XPS being a surface analysis *vs.* XRD looking at the bulk properties of the material. SpEC-Fe5% and 20% show a superficial Fe content of ∼0.10 at. %, comparable to that of Sp-Fe10%. This indicates that within 5%–20% Fe Fe loadings, the formation of superficial Fe species, mainly Fe° and Fe carbide, remains in a thermodynamic equilibrium while metallic Fe evolves to Fe_3_C.^50^ Interestingly, the concentration of surface Fe species increases up to ∼0.40 at. % in the SpEC-Fe50%, which correlates with EDS chemical analysis (Figure S2e), indicating the agglomeration of Fe species on the surface of the catalyst.

For further investigation of the chemical environment on the surface of these materials, C 1s peak fitting was performed (Figure 6b), placing the peak center of C 1s ∼ 284.4 eV, corresponding to C=C bonding.^51^ From the fitting process, all the samples show a dominant C=C peak correlated with a graphitic-like structure. Nonetheless, C–C low binding energy defective domains (∼284 eV) and C–C high binding energy defective domains, which overlap with adventitious C (∼284.8 eV), were fitted. For SpEC-Fe*x* samples with different Fe(OAc)_2_ ratios, C 1s peak fitting shows a dominant signal around the tail feature of the spectra, corresponding to C–O (∼286.2 eV) and COO (∼288.9 eV), which correspond to the expected remaining oxygen functionalities of SpECs polymeric structure (C–OH and O=C–OH).^51^ Additionally, O 1s high-resolution spectra analysis confirms the dominant presence of C–O functionalities and the presence of SiO_2_ and CaO in agreement with the findings of the XRD characterization (see Section 3.2).^51^ Finally, the Fe 2p high-resolution spectrum was fitted, encountering the expected Fe°, Fe^2+^ 2p^3/2^, and Fe^3+^ 2p^3/2^ centered at ∼706.9, 709.6, and 711.51 eV, respectively. These findings provide additional evidence of the formation of Fe° particles embedded in the carbonaceous matrix and the presence of Fe_3_C correlated with the Fe^2+^ 2p^3/2^ peak. The presence of Fe^3+^ 2p^3/2^ suggests the oxidation of Fe_3_C, which can be attributed to the slow cooling down process in the tubular furnace and the exposure to air. In particular, the low signal from Fe^3+^ 2p^3/2^ and satellite shake-ups imply oxidation and spin–orbital hybridization of these species.^50^

### 3.7 Proposed Mechanism and Evaluation of Carbon Structure

The formation of SpEC-supported graphene-encapsulated iron catalysts starts with the dispersion of Fe nanoparticles on the surface of the SpECs *via* impregnation. According to the FTIR results (Figure S9), the surface of SpECs is rich in hydroxyl groups. The hydroxyl groups have been shown to chelate the Fe ions and help alleviate the agglomeration of Fe atoms,^52^ as shown by the SEM and EDS results (Figures 3 and S2, respectively).

Numerous studies have attempted to describe the complex reaction between the carbon and the Fe precursor throughout the high-temperature reduction process.^53−55^ The reaction has been believed to occur via gaseous phase interaction with CO and H_2_ and not necessarily with the carbon material itself.^55^ CO and H_2_ are produced via carbon gasification with CO_2_ and H_2_O at high temperatures (>800 °C). The produced CO and H_2_ help reducing the Fe species (usually ferric oxide (Fe_2_O_3_)) to Fe_3_O_4_ first at temperatures above 570 °C, followed by its reduction to FeO between the temperatures of 670 and 870 °C. Subsequently, FeO is reduced to metallic Fe (α-Fe) mainly by the presence of CO and H_2_, which are constantly being produced by the gasification of carbon at high temperatures.

From the analysis of the above characterization results, the encapsulation of Fe_3_C/Fe particles by few-layered graphene sheets can be explained as a synergistic effect of high-temperature self-assembly growth and carbon dissolution–precipitation.^31,56^ At a temperature of 1000 °C, the α-Fe gets transformed into γ-Fe.^31^ The attraction force of the unpaired electrons between 3d of Fe and 2p of carbon dominates; simultaneously, the carbon atoms start dissolving into the metallic Fe particles.^56^ As the carbon atoms start dissolving, they take up openings that are present between the Fe atoms; therefore, this progression pushes the Fe atoms apart. Once the amount of available carbon becomes saturated, the excess carbon leads to the new metastable phase of Fe_3_C. This results in a core composed of a two-phase mixture of metallic Fe (γ-Fe) and Fe carbide (Fe_3_C). This is the reason why the XRD pattern of SpEC-Fe50% showed predominately metallic Fe patterns (Section 3.2), as there was not enough carbon available which could bind with Fe to form Fe_3_C. The encapsulation process starts as the temperature starts to cool down. The nucleation of carbon species is initiated by precipitation of carbon atoms over the surface of metallic Fe, thus reducing their surface energy. Nucleation and growth occur via the continuous deposition of carbon over the bare Fe surface. The entire particle would be covered with an extended sp^2^ carbon network (containing hexagons, pentagons, and islands) and would develop along the tangent direction of the surface of the Fe/Fe carbide particle and be accumulated via a graphitic shell. Further crystallization and growth of multilayer graphitic encapsulation of Fe particles is encouraged. This occurs when the kinetic energy is not sufficient to lift the carbon island off the surface of the Fe particle; hence, the size of the island increases until the particle is fully enclosed.^31^ The overall effect that leads to the formation of encapsulated stable α-Fe/Fe_3_C particles by graphite can be inferred through the XPS surface analysis (Figure 6); the C 1s high-resolution spectrum analysis strongly suggest that the α-Fe metallic particles, as well as Fe_3_C crystals, enhance graphitization during thermal treatment and get encapsulated during the process. This is correlated with a clear dominance of the C sp^2^ peak of this spectrum. Furthermore, the analysis of the Fe 2p high-resolution spectrum supports the proposed mechanism, showing a clear formation of metallic Fe prior to the formation of the carbide phase. Nonetheless, the presence of Fe^3+^ species implies the unsuccessful encapsulation of some Fe species during thermal treatment. This remaining surface Fe species, mainly metallic and carbide, without a protective graphitic layer, can easily oxidize to a more thermodynamically stable phase during the annealing and cooling down processes of the tubular furnace and, finally, the interaction with air post pyrolysis.

### 3.8 Catalysis Application

There is a demanding requirement to reduce atmospheric carbon dioxide (CO_2_) emissions, as it is the most impactful greenhouse gas toward anthropogenic climate change.^57^ Therefore, huge importance has been given to reactions that utilize CO_2_ by scientists all over the world. The RWGS reaction is an example of such a procedure, which utilizes CO_2_ by reacting it with hydrogen (H_2_) to form carbon monoxide (CO) and water vapor (H_2_O). RWGS is an excellent method for converting CO_2_, as the obtained CO can be further transformed into valuable chemicals and fuels *via* sophisticated gas conversion technologies like the Fischer–Tropsch (FT) process and methanol synthesis.^58^

Unfortunately, the conversion of CO_2_ on a large industrial scale is very difficult due to its stable nature. The RWGS process is an endothermic reaction. Hence, it requires high temperatures and, therefore, a substantial input of heat to produce CO. Additionally, the reaction can also produce less valuable byproducts such as CH_4_ via the Sabatier reaction, which occurs through an analogous process taking place in the low-medium temperature range. Noble metals like Pt have been successfully utilized as catalysts in the RWGS reaction;^59^ however, they tend to be either scarce and/or prohibitively expensive. Thus, the present challenge is to develop active, selective, and stable alternative RWGS catalysts based on first-row transition metals.

Various transition metals, including Cu-, Zn-, Co-, and Ni-based catalysts, have proven to be effective for the RWGS reaction. Nevertheless, the long-term stabilities of these catalysts at high temperatures have proven to be very poor.^60−62^ Furthermore, these transition metal catalysts produce methane (via the Sabatier reaction) or suffer rapid deactivation, specifically when CO is one of the obtained products.^63^ An extensive investigation of iron-based catalysts has been reported primarily on the water–gas shift reaction;^64^ however, very few studies exist on RWGS reactions over Fe-based catalysts. Nearly all the works that study Fe-based catalysis for RWGS reactions, involve complex and expensive methods of synthesis.^65,66^ Moreover, these reported catalysts utilize synthetic and/or metal-based catalyst supports, which also require promoters.

**Figure 7:**
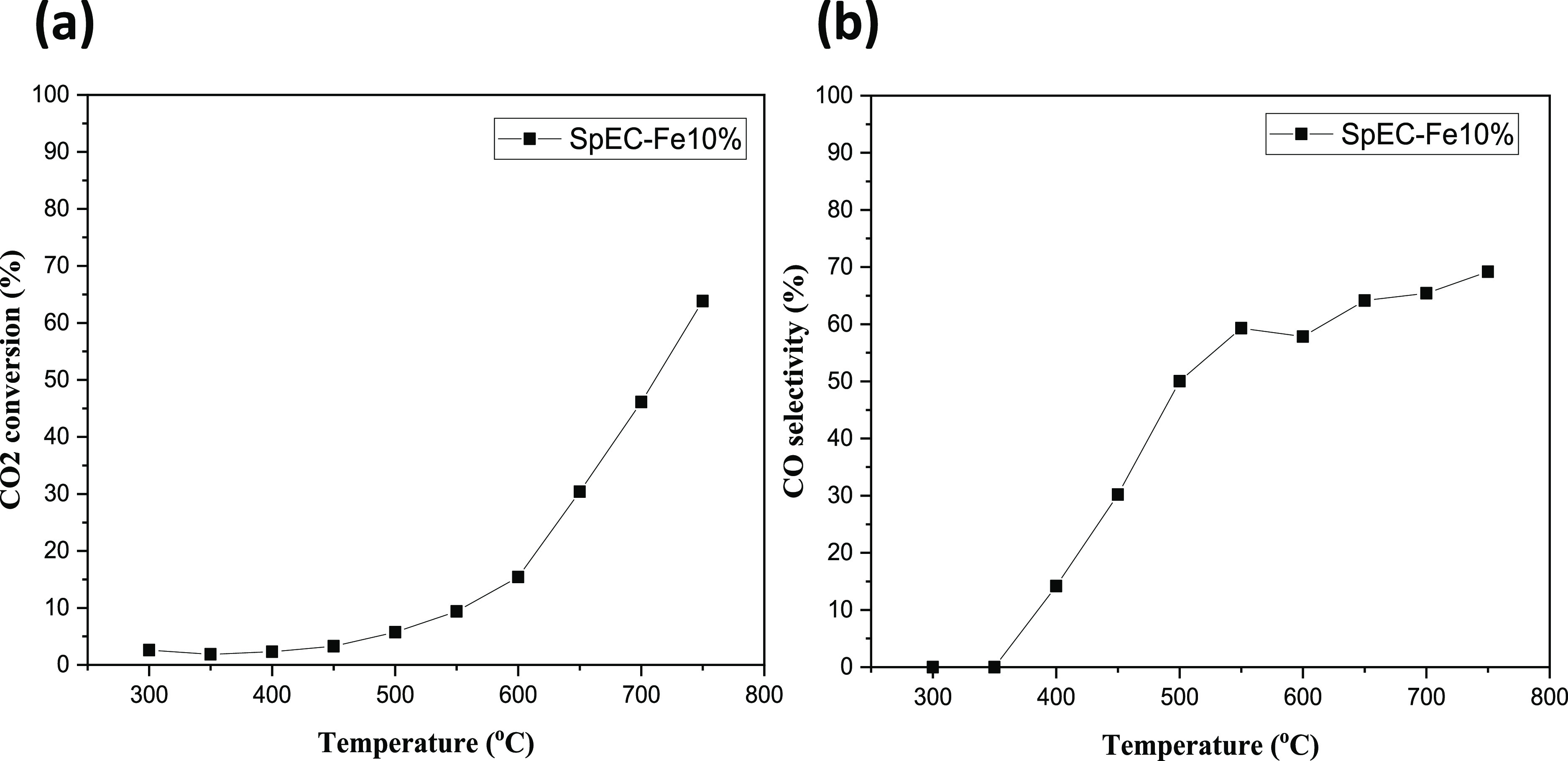
CO_2_ conversion (a) and CO selectivity (b) of the SpEC-Fe10% composite. Conditions: H_2_/CO_2_ = 4:1, WHSV = 12,000 mL g^–1^ h^–1^, and *T* = 300–750 °C.

In our case, the SpEC-Fe10% composite was chosen to be assessed as a potential catalyst for the RWGS reaction. Figure 7 shows the CO_2_ conversion and CO selectivity during the catalytic reaction. CO_2_ conversion levels clearly rise with an increase in temperature, confirming the endothermic nature of the RWGS reaction. A similar trend can be seen in terms of CO selectivity, where an increase is observed with rising temperatures. Importantly, CH_4_ was not detected during the reaction, indicating that the Sabatier reaction was not taking place.

**Figure 8:**
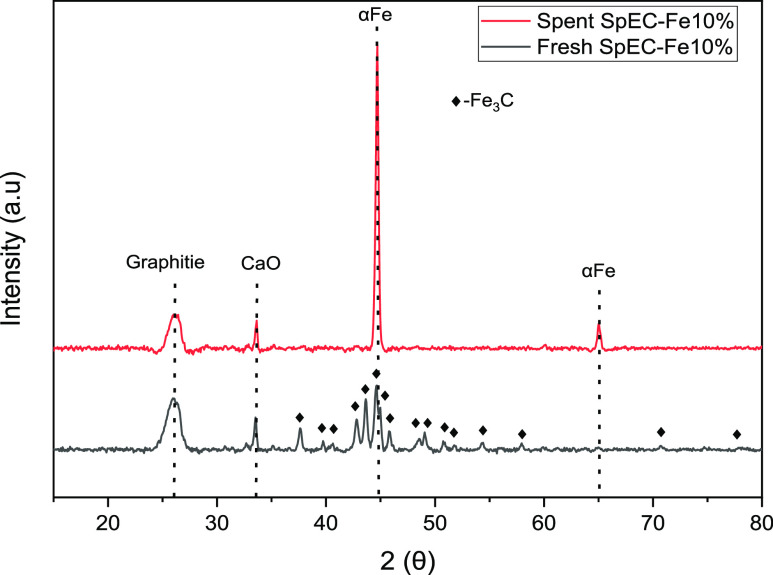
XRD diffraction patterns of the fresh and spent catalysts postreaction.

The XRD patterns of the fresh and spent samples after the RWGS reaction are shown in Figure 8. The iron carbide phase from the fresh sample was nearly all transformed to the metallic iron phase, which most likely occurred due to the reducing environment during the RWGS reaction. Zhang and co-workers^66^ demonstrated that metallic Fe phase is more active than iron carbide for the RWGS reaction, this supports the good catalytic performance reported here. While more evidence is needed with a more in-depth discussion on the changes in catalyst structure and morphology, the fact that our catalyst retained and actually increased its metallic iron phase provides initial clues that our catalyst will show good stability. Furthermore, the retention of the graphite peak in the spent catalyst suggests that the encapsulated metallic iron particles remain protected, which is a potential further indication of good catalyst stability.

It is also worth mentioning that the morphology (surface area, size, and distribution of pores) may change during catalysis, which may affect the catalyst performance. While this is the object of further work being carried out by the same authors, Zhang and co-workers^66^ found the changes in textural properties (porosity and surface area) not to be the main determining factors in the RWGS reaction.

## 4 Conclusions

Since the structure of SpECs is different from conventional lignocellulosic biomass, our aim was to utilize SpECs as a catalyst support for Fe. We demonstrated an efficient method of synthesizing sustainable CEINP. Few-layered graphene sheet encapsulated Fe particles were successfully synthesized by high-temperature pyrolysis (N_2_, 1000 °C) of Fe-treated SpECs. The synthesized SpECs-supported Fe particles characteristically comprised of α-Fe/Fe_3_C core–shell encapsulated by graphitic carbon layers. Our results clearly demonstrate that the porosity, surface area, and degree of graphitization are easily tunable with the amount of Fe loading on our proposed support. Ordered graphitic layers were observed with an increase in Fe loading and in the SpECs sample with no Fe. Significantly, the ability to replicate the morphology and structure of the untreated SpECs was demonstrated with a high degree of precision even after high amounts of Fe loading (up to 32.2 mg of Fe-20% by weight of Fe(OAc)_2_). Fe loading exceeding 20% by weight using Fe(OAc)_2_ produced a SpEC matrix that lost its original morphology, with aggregation of excess Fe being observed. The SpECs with no Fe loading also produced graphitic domains and were also able to retain the original structure and morphology, even after the high-temperature treatment, showing off their high thermal stability. This study established the role of SpECs as an effective biomass waste-based natural-origine carbon support for producing graphite-encapsulated Fe particles.

To test the ability of the synthesized composite, the SpEC-Fe10% sample was chosen to be assessed as a potential catalyst for the reverse RWGS reaction. Preliminary results demonstrated that the catalyst is highly active and selective for the RWGS reaction. Unlike the majority of the catalysts being developed for RWGS reactions, our catalyst has demonstrated that it is possible to obtain high activity and selectivity with a very sustainable and inexpensive method. Additionally, we were able to obtain promising results without the need for a promoter, which is also a significant benefit in terms of cost and ease of synthesis. These are, however, only initial results, and the catalyst will need to be tested comprehensively for the RWGS reaction. In addition, and for completeness of the argument, we should highlight that, while introducing significant advantages such as thermal and chemical stability and uniformity of shape, the use of SpECs as catalysis support carries a slight disadvantage when compared to any other biomass type. SpECs require to be extracted from Sp particles typically, via an acid or alkaline wash, and while this is a consolidated procedure, this is an extra step in the preparation that other types of biomass would potentially not require. Nevertheless, this opens the door for SpECs to be utilized as catalyst support for other potential applications in various catalytic systems, including Fischer–Tropsch, oxygen reduction reactions, and many sustainable processes. Even though pollen exines were utilized in this study, there are numerous plant species that reproduce pollen grains, with each pollen grain having its own unique carbonaceous matrix morphology and structural composition. Hence, further investigation should be conducted, as not enough research has been done on employing pollen grains as catalyst supports in heterogeneous catalyst systems.

## Supporting Information Available

EDS Elemental analysis of untreated sample, SEM images, Raman spectra and XPS spectra of treated samples and FTIR spectrum of untreated sample (PDF)
