# Supplementary material for: Synthesis of a Graphene-Encapsulated Fe3C/Fe Catalyst Supported on Sporopollenin Exine Capsules and Its Use for the Reverse Water–Gas Shift Reaction
Source: ACS Sustain Chem Eng. 2023 Oct 21;11(44):15795–807. doi: 10.1021/acssuschemeng.3c00495 (PMC10630965; doi:10.1021/acssuschemeng.3c00495)
Supplement: Supplementary file 1 — sc3c00495_si_001.pdf [file sc3c00495_si_001.pdf]

Electronic supplementary information

Synthesis of a Graphene-Encapsulated Fe_3C/Fe Catalyst Supported on Sporopollenin Exine Capsules, and Its Use for the Reverse Water-Gas Shift Reaction

Waqas Malik[a], Jorge Pavel Victoria Tafoya[a], Szymon Doszczeczko[a], Ana Belen Jorge Sobrido[a], Vasiliki K. Skoulou[b], Andrew N. Boa[b], Qi Zhang[c], Tomas Ramirez Reina[c] and Roberto Volpe[a*]

[a]School of Engineering and Materials Science, Queen Mary University of London, Mile End Campus, E1 4NS, London, United Kingdom

[b]Department of Chemistry, University of Hull, Hull, HU6 7RX, UK

[c]Department of Chemical and Process Engineering, University of Surrey, Guildford, Surrey GU2 7XH, UK

[*] Corresponding Author. Tel: +44 (0)20 78827748. E-Mail: r.volpe@qmul.ac.uk,

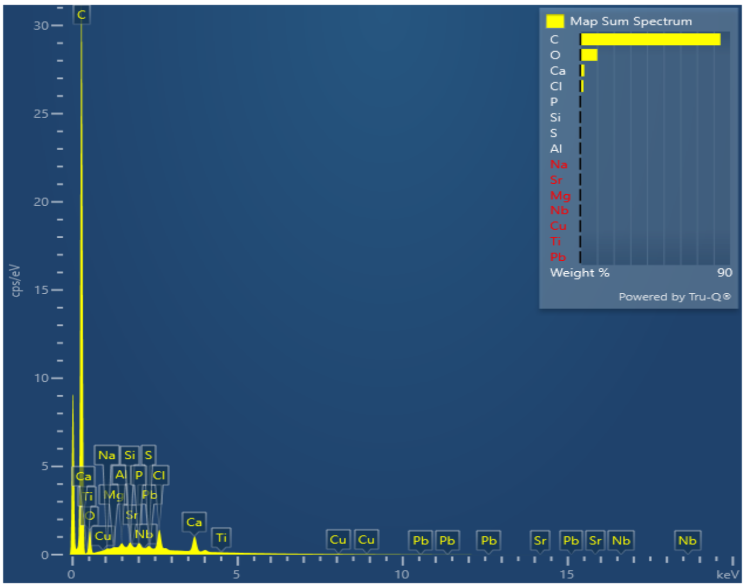

Figure S. 1 - EDS elemental analysis of untreated SpEC

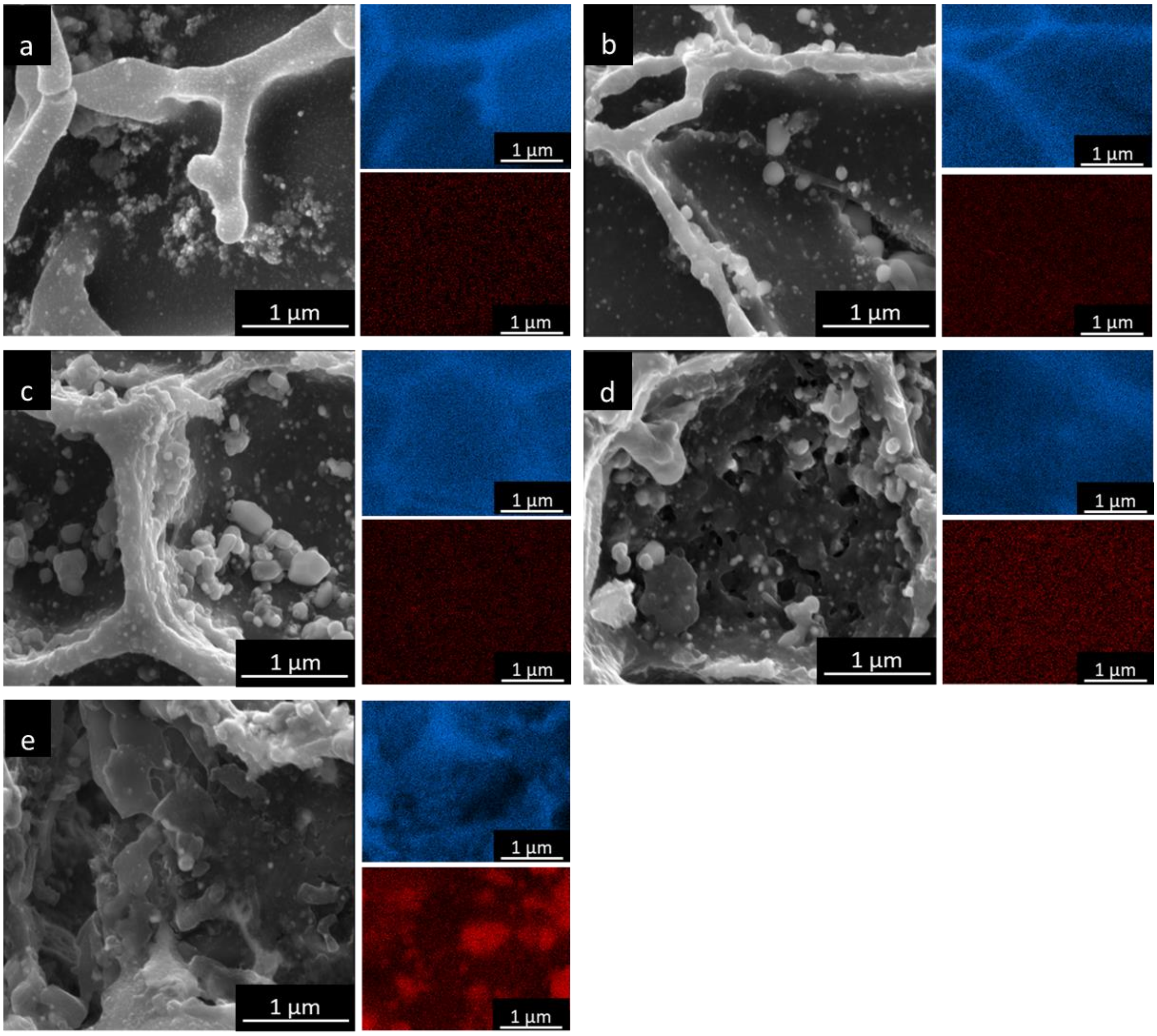

Figure S. 2- SEM images and corresponding EDS mapping of SpEC-Fex (Blue map indicates carbon signal and red map indicates Fe signal). (a) SECp-Fe1%, (b) SpEC-Fe5%, (c) SpEC-Fe10%, (d) SpEC-Fe20% and (e) SpEC-Fe50%.

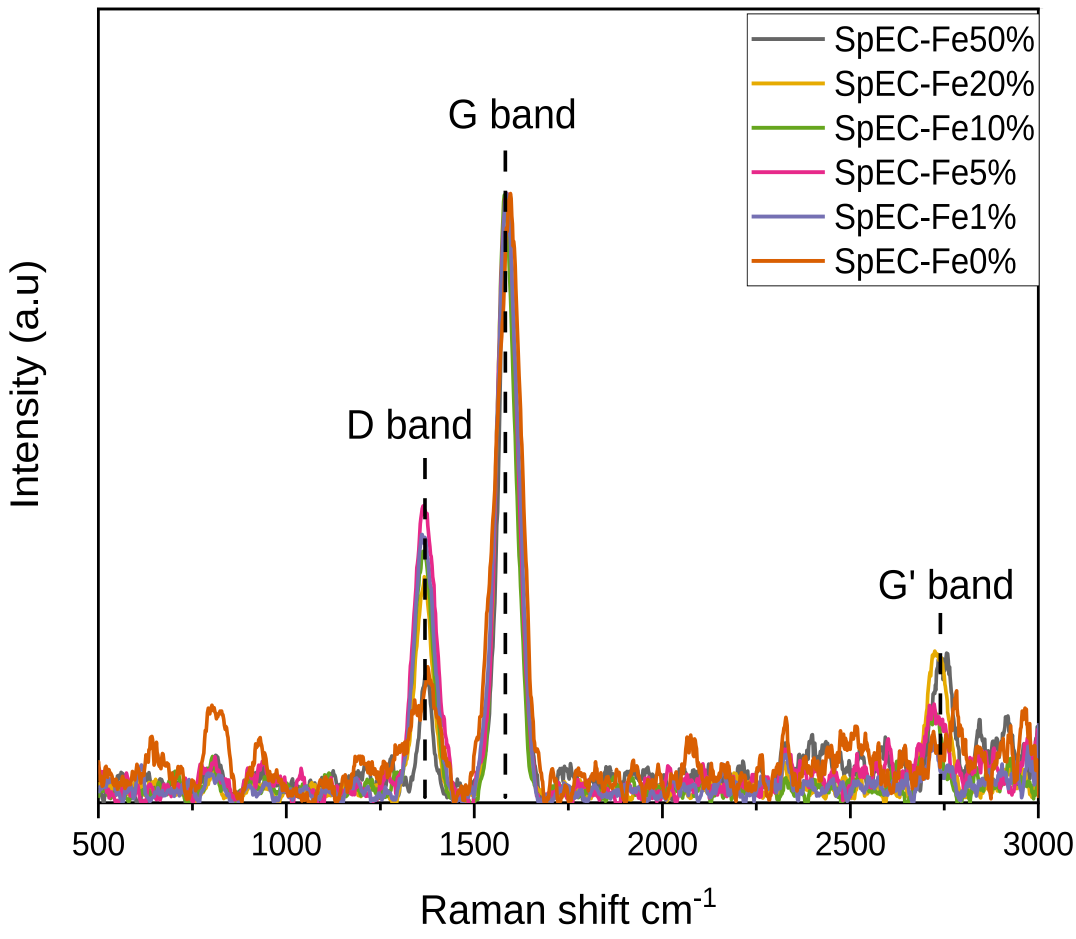

Figure S. 3- Raman spectra depicting the D and G and G' bands of all SpEC-Fex composites.

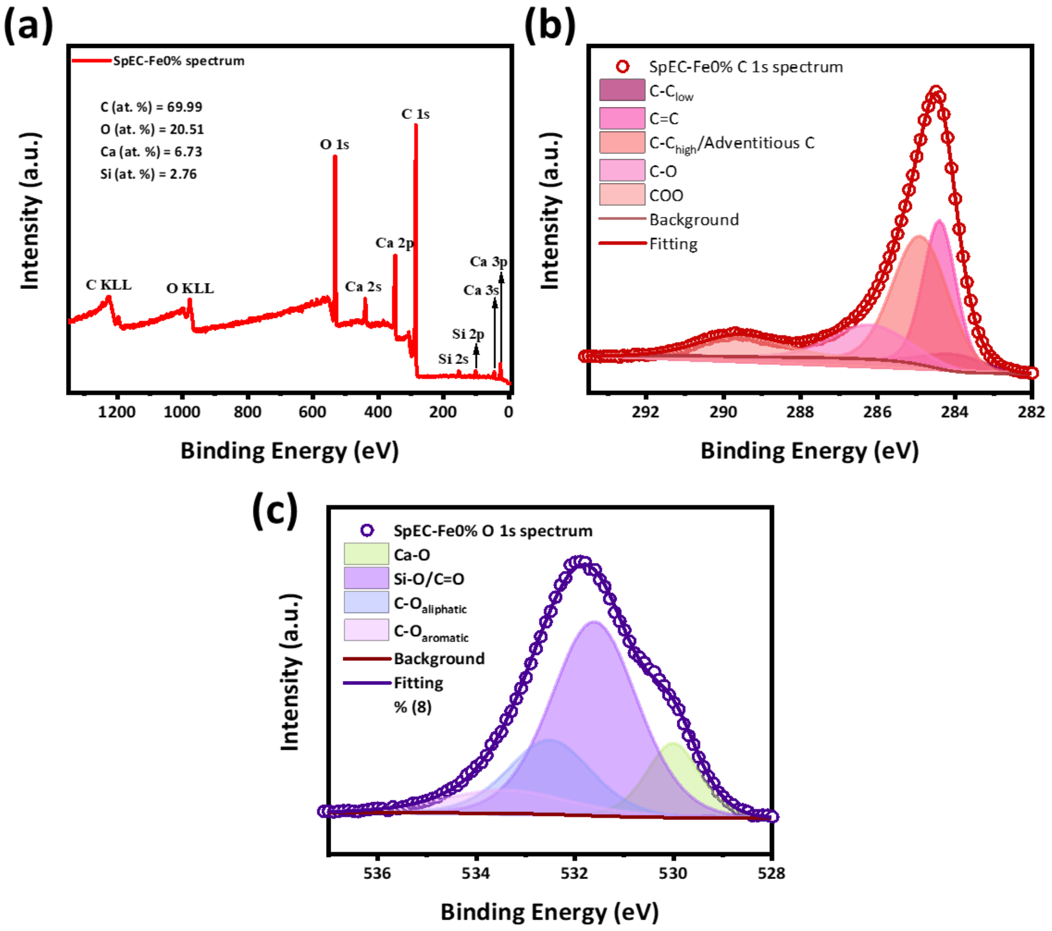

Figure S. 4-X-ray photoelectron spectra analysis of SpEC-Fe0% sample: (a) Survey spectrum, (b) C 1s high resolution spectrum, (c) O 1s high resolution spectrum

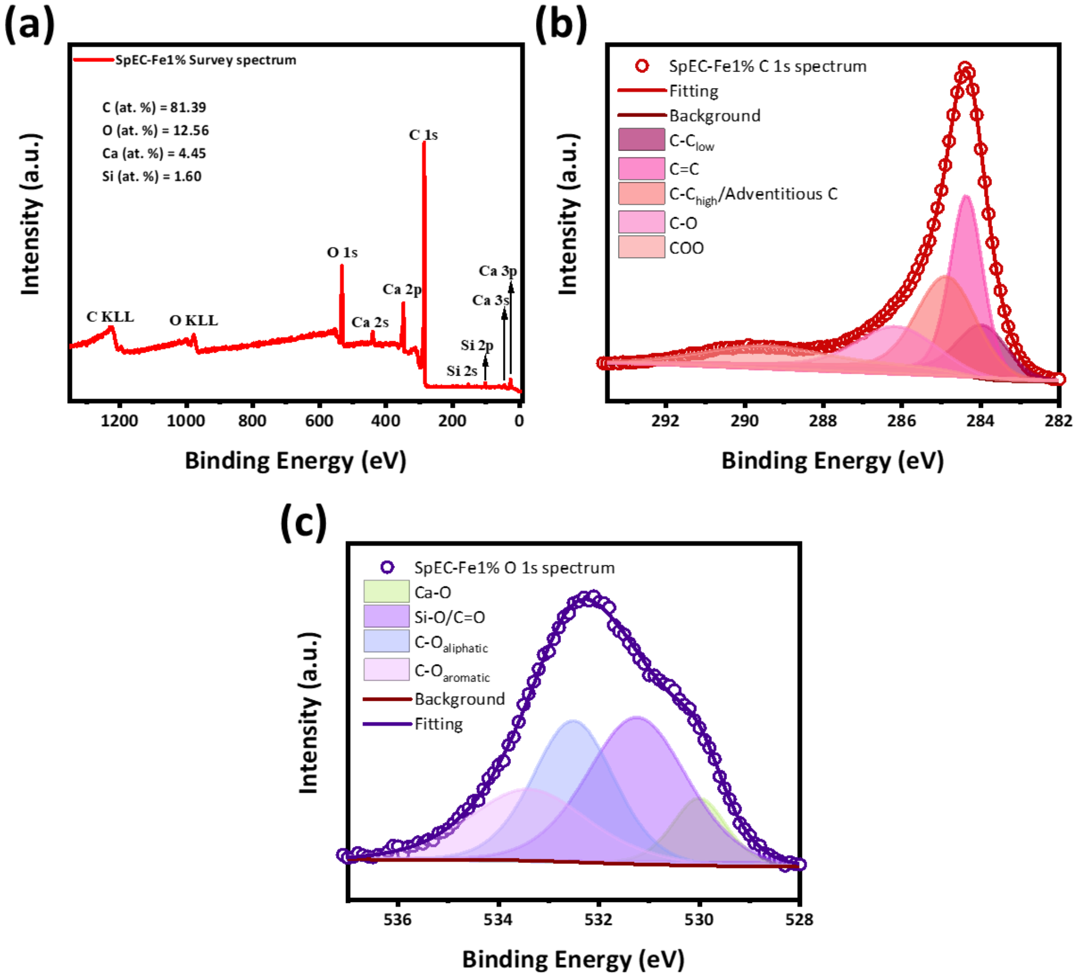

Figure S. 5-X-ray photoelectron spectra analysis of SpEC-Fe1% sample: (a) Survey spectrum, (b) C 1s high resolution spectrum, (c) O 1s high resolution spectrum

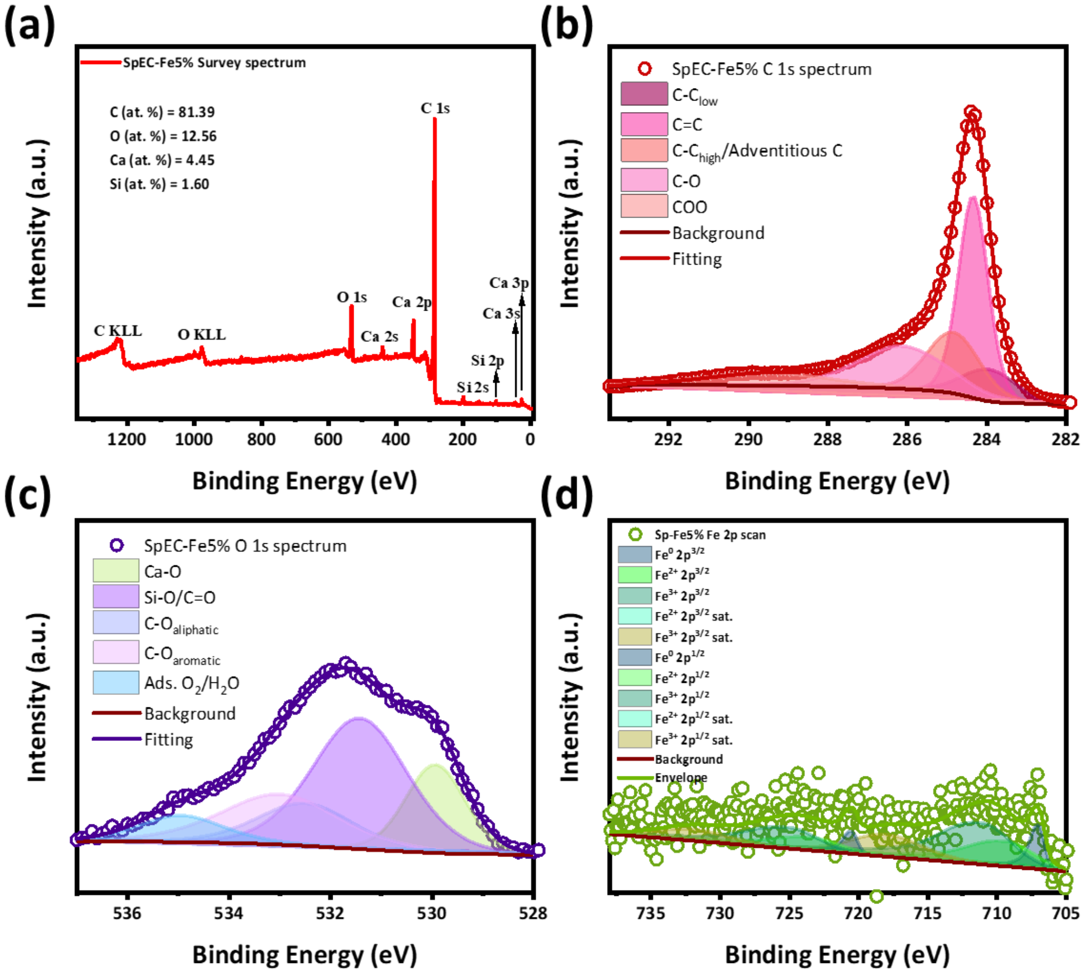

Figure S. 6 -X-ray photoelectron spectra analysis of SpEC-Fe5% sample: (a) Survey spectrum, (b) C 1s high resolution spectrum, (c) O 1s high resolution spectrum and (d) Fe 2p high resolution spectrum

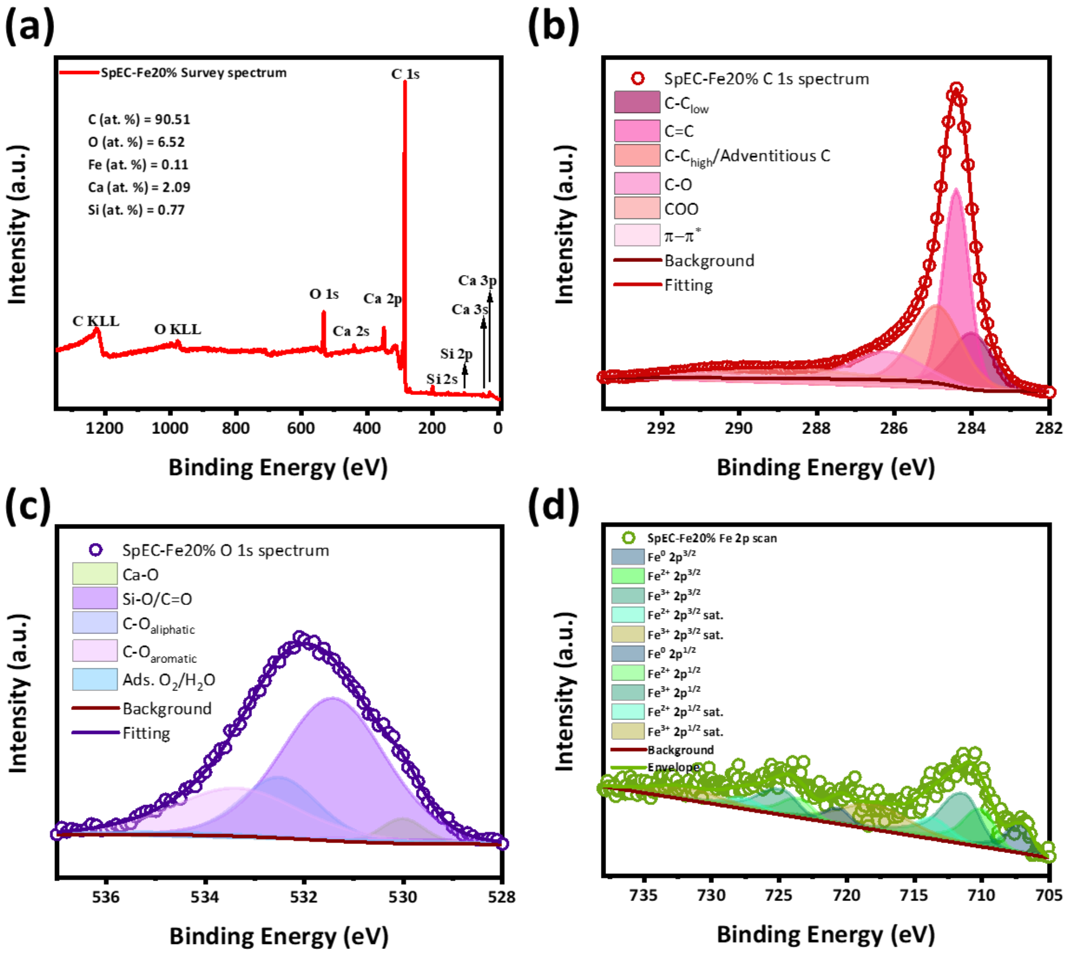

Figure S. 7 – X-ray photoelectron spectra analysis of SpEC-Fe20% sample: (a) Survey spectrum, (b) C 1s high resolution spectrum, (c) O 1s high resolution spectrum and (d) Fe 2p high resolution spectrum

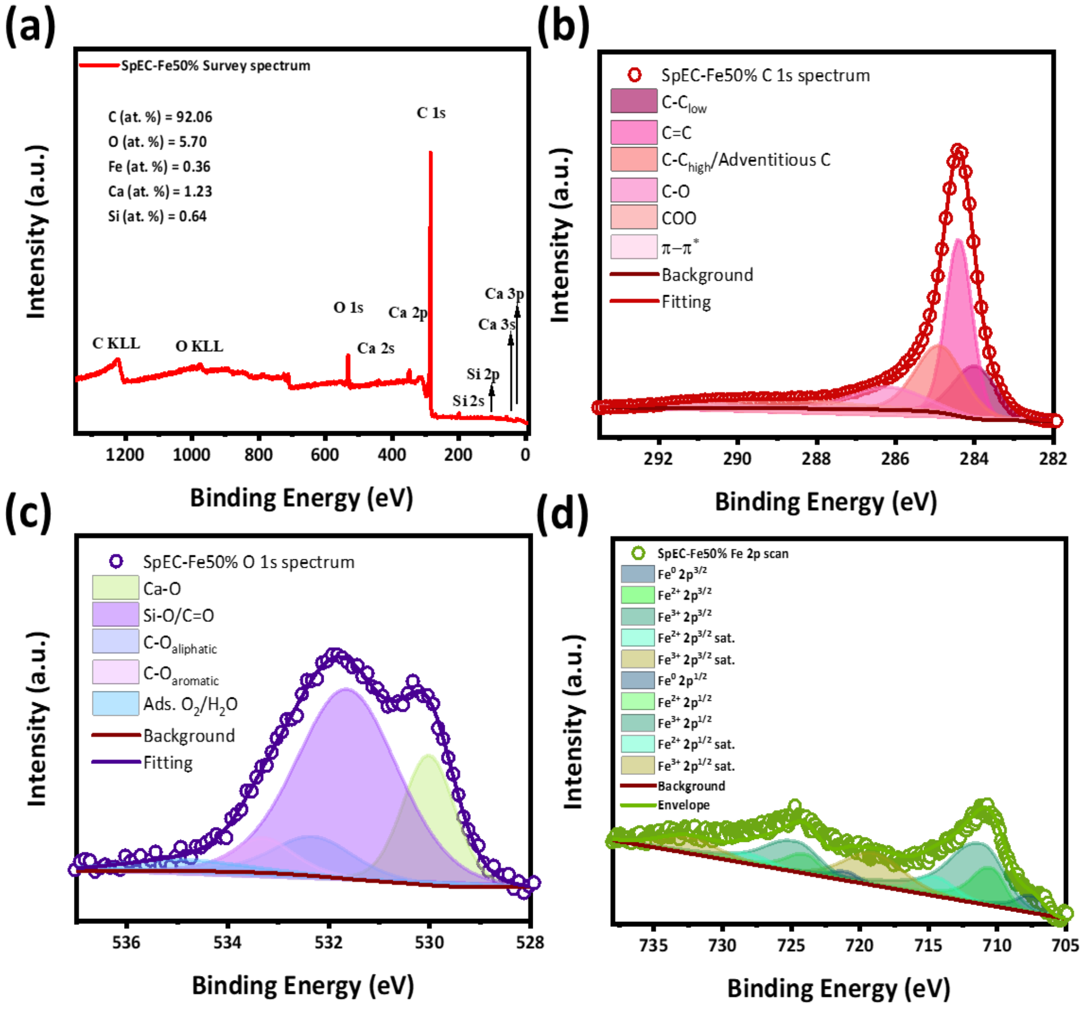

Figure S. 8 -X-ray photoelectron spectra analysis of SpEC-Fe50% sample: (a) Survey spectrum, (b) C 1s high resolution spectrum, (c) O 1s high resolution spectrum and (d) Fe 2p high resolution spectrum

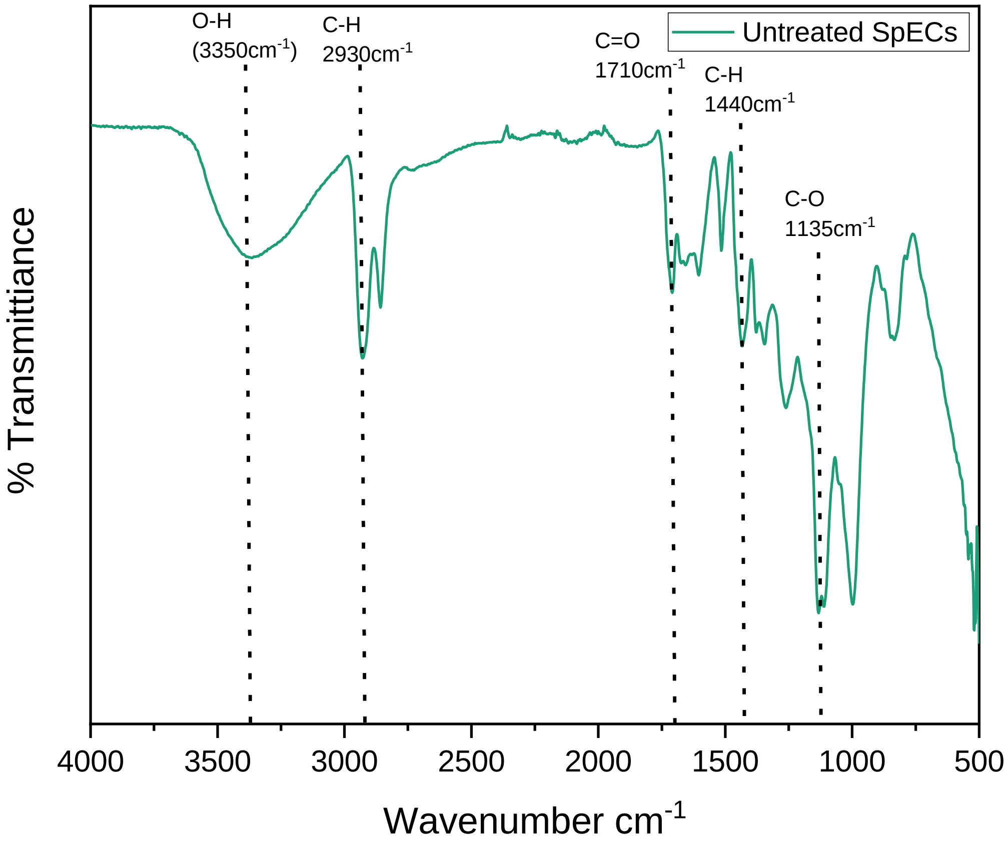

Figure S. 9 -FT-IR spectrum of untreated SpEC.
